# Identification of CSPG4 as a Biomarker and Therapeutic Target for Infantile Post‐Hemorrhagic Hydrocephalus via Multi‐Omics Analysis

**DOI:** 10.1002/advs.202410056

**Published:** 2024-12-16

**Authors:** Juncao Chen, Lin Wang, Xiangwen Peng, Tingting Cheng, Yihui Yang, Jingzhen Su, Hongmei Zou, Siyao Wang, Yueting Mao, Lixiang Wu, Xuntao Yin, Minxu Li, Mingwei Zhu, Wei Zhou

**Affiliations:** ^1^ Department of Neonatology Guangzhou Women and Children's Medical Center Guangzhou Medical University Guangzhou 510623 China; ^2^ Department of Radiology Guangzhou Women and Children's Medical Center Guangdong Provincial Clinical Research Center for Child Health Guangzhou 510623 China; ^3^ Institute of Pediatrics Guangzhou Women and Children's Medical Center Guangzhou Medical University Guangzhou 510623 China; ^4^ Changsha Hospital for Maternal and Child Healthcare Changsha 410100 China; ^5^ Department of Neonatology Dongguan Maternal and Child Health Hospital Dongguan 523057 China; ^6^ Key Laboratory of Developmental Disorders in Children Liuzhou Maternity and Child Healthcare Hospital Liuzhou 545006 China

**Keywords:** cerebrospinal fluid, CSPG4, machine learning, post‐hemorrhagic hydrocephalus, proteomics and metabolomics analysis

## Abstract

Intraventricular hemorrhage in preterm neonates has become a major global health problem and is associated with a high risk of post‐hemorrhagic hydrocephalus (PHH). Identifying diagnostic markers and therapeutic targets is a focal challenge in the PHH prevention and control. Here, this study applies multi‐omics analyses to characterize the biochemical, proteomic, and metabolomic profiles of the cerebrospinal fluid (CSF) in clinical human cohorts to investigate disease development and recovery processes occurring due to PHH. Integrative multiomics analysis suggests that the over‐representation of ferroptosis, calcium, calcium ion binding, and cell adhesion signaling pathways is associated with PHH. Bioinformatic analysis indicates that chondroitin sulfate proteoglycan 4 (CSPG4) is discovered as a CSF biomarker and positively correlated with the ventricular size and the rate of periventricular leukomalacia. Next, it is further demonstrated that these signaling pathways are dysregulated in the choroid plexus (ChP) in PHH by using in vitro cellular experiments and rat models of PHH, whereas *CSPG4* silencing can suppress ferroptosis, cell adhesion function, and the intracellular flow of Ca^2+^. These findings broaden the understanding of the pathophysiological mechanisms of PHH and suggest that CSPG4 may be an effective therapeutic target for PHH.

## Graphical abstract

This study demonstrates that over‐representation of ferroptosis, calcium, calcium ion binding, and cell adhesion signaling pathways in cerebrospinal fluid is associated with infantile post‐hemorrhagic hydrocephalus (PHH). Importantly, the expression level of CSPG4 is correlated with PHH progression; and *CSPG4* silencing suppresses the dysregulation of these pathways in PHH models, suggesting that CSPG4 is a biomarker and therapeutic target for PHH.

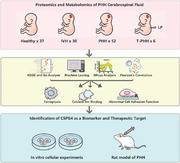

## 1 Introduction

Germinal matrix hemorrhage–intraventricular hemorrhage (GM–IVH) is the most common neurological disease in preterm infants, affecting millions of infants worldwide and having a strikingly increased incidence at lower gestational ages.^[^ ^1^ ^]^ Population‐based studies have shown that ≈20% of premature and very low birth weight newborns developed GM–IVH.^[^ ^2^, ^3^ ^]^ Despite advances in infant intensive care in recent decades, ≈50–75% of infants who survive GM–IVH still develop neurologic sequelae and have a 20–30% mortality rate in the United States.^[^ ^4^, ^5^ ^]^

Up to one‐half of preterm infants with high‐grade GM–IVH (Grades III and IV) progress to posthemorrhagic hydrocephalus (PHH).^[^ ^6^, ^7^ ^]^ Of them, 85% experience cognitive deficits, and 70% suffer motor deficits. Severe PHH can cause acute brainstem herniation and subsequent death.^[^ ^6^, ^7^, ^8^ ^]^ Currently, invasive procedures including serial lumbar punctures (LPs), external ventricular drainage, and ventriculoperitoneal shunts are used to manage PHH. Drug treatments for PHH remain unavailable because of a limited understanding of PHH pathogenesis.^[^ ^9^, ^10^ ^]^ It is generally accepted that the etiology of PHH is multi‐factorial and is associated with the disruption of normal cerebrospinal fluid (CSF) production, flow, and absorption.^[^ ^11^, ^12^ ^]^ Meanwhile, previous studies also have confirmed that changes in CSF components can serve as biomarkers for disease detection and are related to disease progression and prognosis.^[^ ^13^, ^14^ ^]^ However, the exact underlying mechanisms remain elusive. Therefore, identifying biomarkers to determine which GM–IVH cases are likely to progress to PHH and exploring the molecular mechanisms underlying PHH are crucial for developing effective therapeutic strategies.

Omics techniques such as proteomics, metabolomics, genomics, and transcriptomics have been widely used to discover the mechanisms of various diseases.^[^ ^15^ ^]^ In recent years, the combination of proteomics and metabolomics has represented a new trend and gained increasing attention. Studies in humans and mice have demonstrated the importance of the CSF proteome and metabolome in understanding the pathogenesis of neurological disorders and hydrocephalus.^[^ ^16^, ^17^ ^]^ However, they usually used small sample sizes, frequently involving more than one etiology, and without matching across age and gender. To date, there have been no reports on the metabolomic and proteomic profiling of the CSF in infants with PHH.

In this study, multi‐omics analyses were applied to characterize the biochemical, proteomic, and metabolomic profiles of the CSF to investigate disease development and recovery processes occurring due to PHH; then using in vitro cellular experiments and a rat model of PHH to verify the muti‐omics results. Importantly, we provided compelling evidence confirming the efficacy of targeting chondroitin sulfate proteoglycan 4 (CSPG4) as a novel treatment for PHH. In summary, this study presents proof‐of‐principle evidence for understanding the pathogenesisof PHH and improving its treatment.

## 2 Results

### 2.1 Calcium, Red Blood Cells, and the Breakdown Products of Red Blood Cells in the CSF are Critical to the Development of PHH

We used a human cohort to investigate the biochemical profiles of CSF samples from PHH infants. In this study, 52 infants with PHH (PHH group), 6 infants with PHH who underwent LPs therapy (T‐PHH group), 30 infants with GM–IVH without hydrocephalus (IVH group), and 37 healthy preterm infants (H group) were included (**Table** **1**). Four groups of research subjects were selected based on their clinical histories and radiological findings (ultrasonography, and/or MRI). With serial LPs treatment following the method in a previous study,^[^ ^18^ ^]^ after serial LP treatment, the size of the lateral ventricle in 6 T‐PHH infants decreased significantly and gradually reverted to normal; these infants had successful treatment outcomes. All patients were premature infants, and none of them had chromosomal aberrations, genetic disorders, or infections of the central nervous system. Table 1 displayed all the infant descriptions along with each infant's data. The median age of 52 PHH infants was 34.8 days. Follow‐up evaluation of PHH newborns ranged from 12 up to 18 months; the incidence of periventricular leukomalacia (PVL) was 71.1% (*n* = 37), and 12‐month mortality was 9.6% (*n* = 5).

**Table 1:** Baseline demographical and routine laboratory characteristics of CSF content among four groups.

Patient's parameters	H group (N = 37)	IVH group (N = 30)	PHH group (N = 52)	T‐PHH Group (N = 6)	*P* value
Gestational age (weeks)^a)^	32.3(3.8)	31.0(4.1)	31.1(4.4)	28.9(1.9)	0.223
Gender (female:male)	16:21	12:18	21:31	1:5	0.950
Age (days)^a)^	12.8(9.2)	24.2(20.7)	34.8(23.1)	52.3(30.2)	0.000
Boby Weight (gram)^a)^	2056(773)	2062(685)	2305(774)	2671(607)	0.098
CSF Investigations					
Whole CSF RBC (10^6^ · L^−1^)^b)^	0(0–100)	1000(0–146000)	3500(1000–1333000)	0(0–2000)	0.000
Whole CSF WBC (10^6^ · L^−1^)^b)^	4(1–29)	4(1–638)	15.5(1–8680)	7(4–27)	0.000
Glucose (mmol · L^−1^)^a)^	3.0(0.7)	2.7(1.2)	1.3(0.7)	1.7(0.4)	0.000
Chloride (mmol · L^−1^)^a)^	122.8(4.7)	120.4(3.9)	118.9(6.2)	119.6(0.5)	0.010
Protein (g · L^−1^)^b)^	1.1(0.6–1.9)	1.4(0.6–3.4)	2.5(0.9–19.8)	1.5(0.9–3.5)	0.000
Calcium (mmol · L^−1^)^b)^	1.5(1.4–1.7)	1.6(1.5–1.8)	1.8(1.4–2.1)	1.7(1.5–1.8)	0.000
Admission characteristics					
Convulsion, No, (%)	NA	0	4(7.69)	0	NA
Altered mental status, No, (%)	NA	0	3(5.77)	0	NA
Respiratory failure, No, (%)	NA	0	9(17.31)	0	NA
Treatment					
Serial lumbar punctures, No, (%)	NA	NA	11(21.15)	6(100)	NA
Ventricular access device, No, (%)	NA	NA	24(46.15)	0	NA
Ventriculoperitoneal shunt, No, (%)	NA	NA	3(5.77)	0	NA
Neurologic sequelae					
PVL, No, (%)^c)^	NA	4(13.3)	37(71.15)	1(16.67)	NA
Death, No, (%)	NA	0	5(9.62)	0	NA

Values are reported as median (min–max range) or number (percentage) or means (standard deviations). CSF, cerebrospinal fluid; H, healthy preterm infants; IVH, Germinal matrix hemorrhage‐intraventricular hemorrhage; PHH, post‐hemorrhagic hydrocephalus; PVL, periventricular leukomalacia; RBC, red blood cell; T‐PHH, post‐hemorrhagic hydrocephalus infants who were given LP treatment; WBC, white blood cell; NA, not applicable.

^a)^ Data are expressed as mean + standard deviation.

^b)^ Data are expressed as median (interquartile range).

^c)^ According to the results of MRI.

CSF samples from the IVH and PHH groups were collected from the first LP before any neurosurgical treatment, whereas samples from the T‐PHH group were collected from the last LP of the serial LP treatment; when the protein had decreased to ≦1.5 g·L^−1^ and erythrocytes to ≦100 mm^−3^ in CSF, the LP was considered as the last LP. The CSF of PHH group had the highest levels of protein (median, 2.5 g L^−1^; range, 0.9–19.8 g L^−1^), calcium (median, 1.8 mmol L^−1^; range, 1.4–2.1 mmol L^−1^), white blood cells (median, 15.5 × 10^6^ L^−1^; range, 1–8680 × 10^6^ L^−1^), and red blood cells (median, 3500 × 10^6^ L^−1^; range, 1000–1333000 × 10^6^ · L^−1^), while had considerably lower level of glucose (mean, 1.3 mmol · L^−1^; standard deviations (SD), 0.7 mmol L^−1^) and chloride (mean, 118.9 mmol · L^−1^; SD, 6.2 mmol · L^−1^), when compared to that of the other three groups (all *P* < 0.05). Notably, protein levels (blood cells break products), calcium levels, and red blood cell counts gradually increased from the IVH to the PHH group, and were down‐regulated in the T‐PHH group. This may indicate that the threshold levels of calcium, red blood cells, and the breakdown products of red blood cells (such as hemoglobin, iron, and bilirubin) in the CSF are critical to the development for hydrocephalus.

### 2.2 The Activation of Calcium ion Binding, Cell Adhesion, and the Oxidoreductase Activity Pathway Correlate with the Progression of PHH

**Figure 1:**
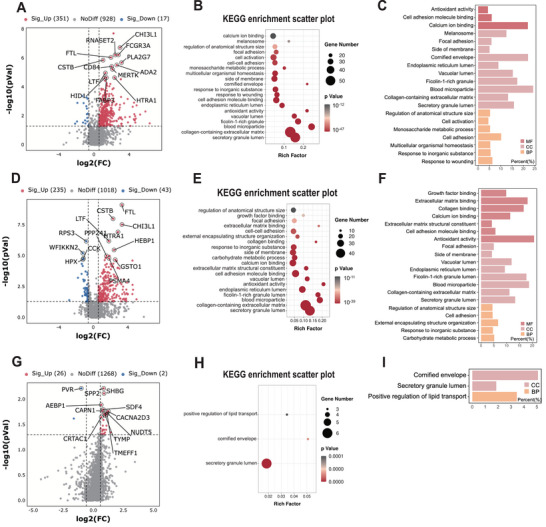
Proteomic alterations occurred in the CSF of PHH infants. A) Volcano plots show the protein alterations in the PHH versus IVH group. B) KEGG‐based enrichment analysis for DEPs in the PHH versus IVH group. C) GO‐based enrichment analysis for DEPs in PHH versus IVH group. D) Volcano plots show the protein alterations in the PHH+ IVH versus H group. E) KEGG‐based enrichment analysis for DEPs in PHH+ IVH versus H group. F) GO‐based enrichment analysis for DEPs in PHH+ IVH versus H group. G) Volcano plots show the protein alterations in the PHH versus T‐PHH group. H) KEGG‐based enrichment analysis for DEPs in PHH versus T‐PHH group. I) GO‐based enrichment analysis for DEPs in the PHH versus T‐PHH group. Note, proteins with |log_2_ (FC)| > 1.5 with an adjusted *P *< 0.05 were considered as potential DEPs in A, D, and G; two‐sided hypergeometric test was used with an adjusted *P *< 0.05 in E and H. H group, healthy preterm infants; IVH, intra‐ventricular hemorrhage infants without hydrocephalus; PHH, post‐hemorrhagic hydrocephalus infants; T‐PHH, PHH infants who had serial LP therapy; BP, biological process; CC, cellular component; MF, molecular function; DEPs, differentially expressed proteins. *n* = 52 for PHH group, *n* = 30 for IVH group, *n* = 32 for H group, *n* = 6 for T‐PHH group.

Label‐free quantitative mass spectrometry (LFQ–MS) was conducted to further elucidate the differential proteins in CSF samples from the four different types of infants (*n* = 125). Compared with the IVH group, 351 differentially expressed proteins (DEPs) were upregulated and 17 DEPs were downregulated in the PHH group (Figure **1**A; and Table S1, Supporting Information). Next, we used Kyoto Encyclopedia of Genes and Genomes (KEGG) pathways and annotations of Gene Ontology (GO) biological processes to map out potential biological processes. The KEGG results showed that the enriched pathways were mainly associated with secretory granule lumen, collagen‐containing extracellular matrix, blood microparticle, ficolin‐1 rich granule, and antioxidant activity (Figure 1B). GO molecular function analysis demonstrated that the DEPs were mainly involved in calcium ion binding, cell adhesion, and antioxidant activity pathways (Figure 1C). A total of 278 DEPs were found between the IVH+PHH and H groups, including 235 upregulated and 43 downregulated proteins (Figure 1D). The enriched KEGG pathways and GO biological processes were similar to those of the PHH versus IVH groups (Figure 1E,F). When compared to T‐PHH infants, the PHH group included 26 upregulated and 2 downregulated proteins (Figure 1G). The results of the KEGG pathways and GO analysis were shown in Figure 1H,I.

CSF proteins varied gradually until disease onset, which could provide clues to the related mechanisms. The H group, IVH group, and PHH group may correspond to the occurrence of PHH in our study. To characterize the expression trends of proteins related to disease progression, DEPs in CSF samples from four groups were divided into 16 patterns (clusters 1– 16) using Mfuzz analysis. As shown in Figures S1 and S2 (Supporting Information), and Table S2 (Supporting Information), we paid more attention to DEPs that changed in the following four patterns in this study: (1) gradually downregulated from H, IVH to PHH group, and then upregulated in the T‐PHH group; (2) gradually upregulated from H, IVH to PHH group, then downregulated in the T‐PHH group; (3) upregulated in both the IVH and PHH groups; and (4) downregulated in both the IVH and PHH groups. Patterns (1) and (2) could reveal the pathogenesis of the disease, whereas patterns (3) and (4) were correlated with disease severity.The results showed that pattern (1) included: albumin, alpha‐fetoprotein, neural epidermal growth factor‐like 2 (NELL2), *etc* (Figure S1: Cluster 1; Table S2, Supporting Information); pattern (2) included: apolipoprotein, CSPG4, heat shock protein 90 alpha family class B member 1(HSP90AB1), *etc* (Figure S1: Cluster 6; Table S2, Supporting Information); pattern (3) was enriched with proteins mainly involved in cell adhesion molecule binding, calcium ion binding, and oxidoreductase activity pathway (Figure S2A, Supporting Information); pattern (4) was enriched with proteins mainly involved in peptidase activity, calcium ion binding, and G protein‐coupled receptor binding pathway (Figure S2B, Supporting Information).

Taken together, these results suggested that activity of calcium ion binding, oxidoreductase activity, and cell adhesion pathway were correlated with the progression of hydrocephalus.

### 2.3 Metabolomic Profiling Reveals Altered Lipid Metabolism During the Progression of PHH

**Figure 2:**
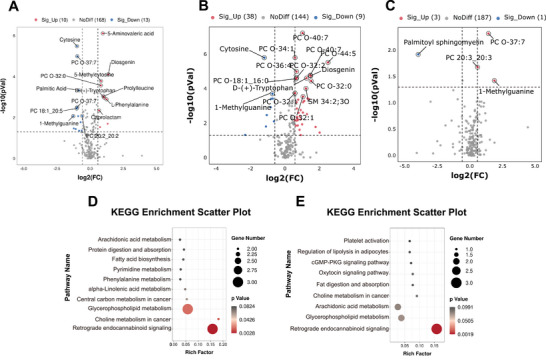
Identification of differentially expressed metabolites (DEMs) in the CSF of from infants with PHH. A) Volcano plots show the metabolites alterations in the PHH versus IVH group. B) Volcano plots show the metabolite alterations in the PHH+ IVH versus H group. C) Volcano plots show the metabolite alterations in the PHH versus T‐PHH group. Metabolites with |log_2_ (FC)| > 1.5 with an adjusted *P *< 0.05 were considered as potential DEMs in A, B, and C. D) KEGG‐based enrichment analysis for DEMs in PHH versus IVH group. E) KEGG‐based enrichment analysis for DEMs in PHH+ IVH versus H group; two‐sided hypergeometric test was used with adjusted *P *< 0.05 in D and E. H group, healthy preterm infants; IVH, intra‐ventricular hemorrhage infants without hydrocephalus; PHH, post‐hemorrhagic hydrocephalus infants; T‐PHH; PHH infants who had serial LP therapy. *n* = 52 for PHH group, *n* = 30 for IVH group, *n* = 32 for H group, *n* = 6 for T‐PHH group.

Hydrocephalus not only impairs brain function, but also disrupts metabolism and consequently resulting in long‐term metabolic disorders.^[^ ^19^, ^20^ ^]^ To examine the global variation in metabolism due to hydrocephalus, an untargeted metabolomic analysis was conducted in CSF samples from four different types of infants (*n* = 125). 10 metabolites were significantly upregulated, whereas 13 metabolites were downregulated in the PHH group compared to the IVH group (Figure **2**A; and Table S3, Supporting Information), and 47 differentially expressed metabolites (DEMs) were found to be significantly differentially expressed in the IVH+PHH group compared to the H group, including 38 upregulated and 9 downregulated metabolites (Figure 2B). Furthermore, we found that 4 metabolites were differentially expressed in the CSF of PHH patients versus T‐PHH patients (Figure 2C). Proteomic and biochemical data showed that many iron and iron metabolism proteins, including ferritin light chain (FTL) and ferritin heavy chain 1 (FTH1), were present at very high concentrations in the CSF (Table 1; Table S1, Supporting Information). Correspondingly, pathway analyses in metabolomic profiling also showed that some metabolites belonged to pathways related to ferroptosis and iron metabolism, such as glycerophospholipid metabolism, fatty acid biosynthesis, and alpha‐linolenic acid metabolism, *etc* (Figure 2D,E).

We also used Mfuzz analysis to characterize the expression trends of metabolites related to disease progression (Figure S2C,D; Table S2, Supporting Information). The results showed that the metabolites involved in glycerophospholipid metabolism, phosphosphingolipid metabolism, and fatty amides were upregulated in both the IVH and PHH groups. Notably, these lipid metabolism pathways have been reported to be associated with ferroptosis.

**Figure 3:**
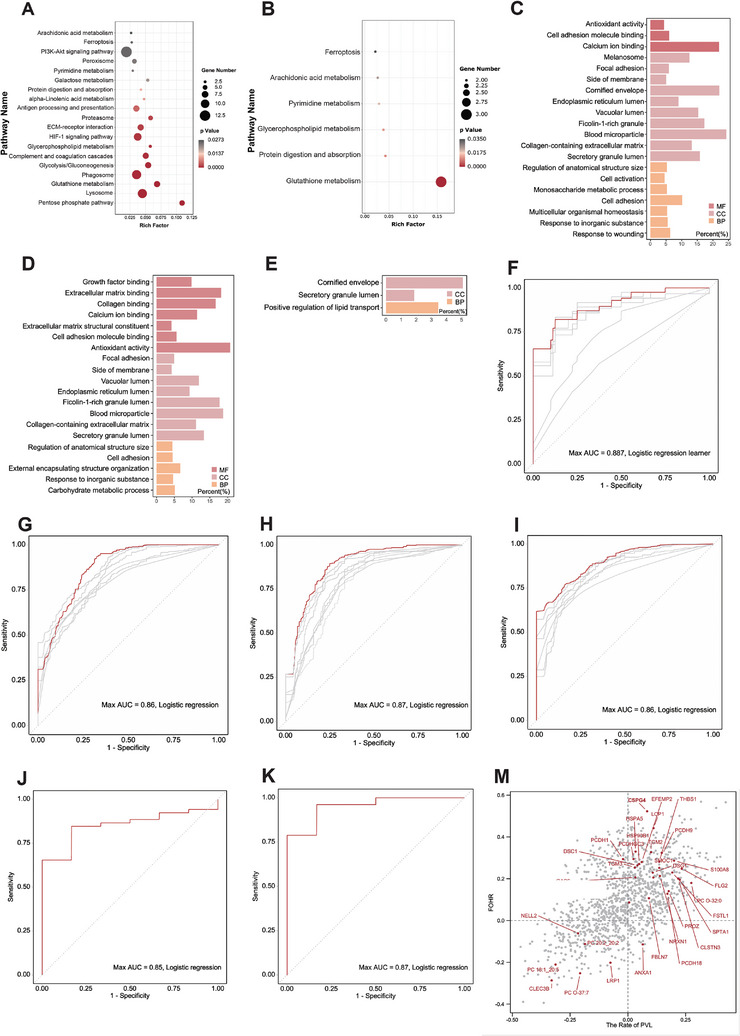
Integration analysis, machine learning, Pearson's correlation analysis of proteomics and metabolomics data. A) KEGG integration analysis of DEPs and DEMs in the PHH versus IVH group. B) KEGG integration analysis of DEPs and DEMs in the PHH+ IVH versus H group. C) GO integration analysis of DEPs and DEMs in PHH versus IVH group. D) GO integration analysis of DEPs and DEMs in PHH+ IVH versus H group. E) GO integration analysis of DEPs and DEMs in PHH versus T‐PHH group. A–E) were completed by the Wu Kong platform (https://www.omicsolution.com/wkomics/main/). F) Machine learning performance of the panel of five protein biomarkers (CDH13, EIF4A1, H6PD, PPP1181, and PPP280) in PHH+ IVH versus H group. Two proteins (PPP1181 and PPP280) could not be found in Uniprot. G) Machine learning performance of the panel of five metabolite biomarkers (PC O‐32:0, cytosine, PC O‐40:7, PC O‐34:1, and PC 18:0_20:5) in the PHH+ IVH versus H group. H) Machine learning performance of the panel of two protein biomarkers (CHI3L1 and SPP1) in the PHH versus IVH group. I) Machine learning performance of the panel of two metabolite biomarkers (cytosine and 5‐Aminovaleric acid) in PHH versus IVH group. J) Machine learning performance of the panel of two protein biomarkers (SPP2 and PVR) in the PHH versus T‐PHH group. K) Machine learning performance of the panel of five metabolite biomarkers (Palmitoyl sphingomyelin, and PC O‐37:7) in the PHH versus T‐PHH group. M) Correlation of CSF proteomic and metabolomic profiles with ventricular size and the rate of PVL using Pearson's correlation. H group, healthy preterm infants; IVH, intraventricular hemorrhage infants without hydrocephalus; PHH, post‐hemorrhagic hydrocephalus infants; T‐PHH; PHH infants who had serial LP therapy. ROC‐AUC: area under the ROC curve. DEMs, differentially expressed metabolites; DEPs, differentially expressed proteins. *n* = 52 for PHH group, *n* = 30 for IVH group, *n* = 32 for H group, *n* = 6 for T‐PHH group.

To gain a holistic view of the significantly altered canonical pathways and molecular interaction networks between PHH and the other three groups, we used the Cytoscape software to integrate proteomic and metabolomic data to identify pathways that related to PHH progression. When comparing the PHH and IVH groups, the top 5 canonical pathways identified were pentose phosphate pathway, lysosome, glutathione metabolism, phagosome, and glycolysis/gluconeogenesis (Figure **3**A). When comparing the PHH+IVH group to the H group, the pathways identified were involved in purine metabolism, arachidonic acid metabolism, pyrimidine metabolism, glycerophospholipid metabolism, protein digestion and absorption, and retrograde endocannabinoid signaling (Figure 3B). The results of the GO analysis were similar to those of the proteomic analysis (Figure 3C–E).

Taken together, the results of metabolomic profiling strongly suggested that the lipid metabolism was altered during PHH progression.

### 2.4 Utilizing Machine Learning and Pearson's Correlation Analysis for the Identification of Proteomic and Metabolic CSF Biomarkers in PHH

Machine learning has been used to accurately classify different subtypes of diseases, and uncover their pathogenesis.^[^ ^21^ ^,^ ^22^ ^]^ Here, we evaluated the predictive power of the validated proteins and metabolites by using machine learning. When distinguishing between the PHH+IVH and H group, the following proteins and metabolites showed the best performance: cadherin 13, eukaryotic translation initiation factor 4A1 (EIF4A1), hexose‐6‐phosphate dehydrogenase (H6PD), PC O‐32:0, cytosine, PC O‐40:7, PC O‐34:1, and PC 18:0_20:5; the maximum area under curve (AUC) values was 0.89 (Figure 3F,G). Importantly, 2 proteins (chitinase 3 like 1[CHI3L1] and secreted phosphoprotein 1 [SPP1]) and 2 metabolites (cytosine and 5‐aminovaleric acid) were successfully identified as the potential biomarkers of PHH infants, discriminating them from the IVH; the total AUC values were 0.86 and 0.87 (Figure 3H,I). As illustrated in Figure 3J,K, SPP2, poliovirus receptor (PVR), palmitoyl sphingomyelin, and PC O‐37:7 were distinguished between the PHH and T‐PHH groups, the maximum AUC was 0.87.

Next, we examined whether the DEPs and DEMs in IVH+PHH infants correlated with ventricular size and the rate of PVL using Pearson's correlation analysis. The results were shown in Figure 3M. We found 2 proteins and 1 metabolite showed a negative correlation (Pearson's correlation coefficient (r) < −0.3, *P* < 0.05); 154 proteins and 8 metabolites showed a positive correlation (r >0.3, *P* < 0.05) with ventricular size. Furthermore, we examined the relationship between the rate of PVL and the DEPs and DEMs in the CSF. 13 proteins and 6 metabolites showed a positive correlation with the rate of PVL, whereas 42 proteins and 5 metabolites showed a negative correlation (Figure 3M). Importantly, 9 proteins and 1 metabolite showed a positive correlation with both the ventricular size and rate of PVL, including SPP1, CH3I1L, CSPG4, and 5‐Aminovaleric acid.

In the above machine learning analysis, CSPG4 was not identified as a potential biomarker to discriminate PHH against IVH. However, considering the results of Pearson's correlation and Mfuzz analysis, we further analyzed the performance of CSPG4 as a potential biomarker for distinguishing PHH from IVH through machine learning. The AUC was 0.79, indicating that CSPG4 can also be used as a potential biomarker for PHH (Figure S3, Supporting Information). Therefore, using a comprehensive approach of machine learning, Pearson's correlation analysis, and Mfuzz analysis, we ultimately chose SPP1, CH3I1L, and CSPG4 as potential biomarkers for PHH, and would make a further validation.

### 2.5 Ferroptosis, Cell Adhesion, and Intracellular Flow of Ca^2+^ were Abnormally activated in the Choroid Plexus in PHH

The production and flow of CSF are completed by choroid plexus (ChP) cells. Thus, CSF abnormalities may indicate an aberrant function of the ChP. Numerous studies have suggested that abnormalities in the ChP cells contributes to PHH.^[^ ^6^, ^11^, ^23^ ^]^ Nonetheless, the biology of ChP cells in PHH remains obscure. Herein, we investigated the biological characteristics of the ChP in PHH using in vitro and in vivo models and analyzed whether the signaling pathway alwas similar to that in CSF.

**Figure 4:**
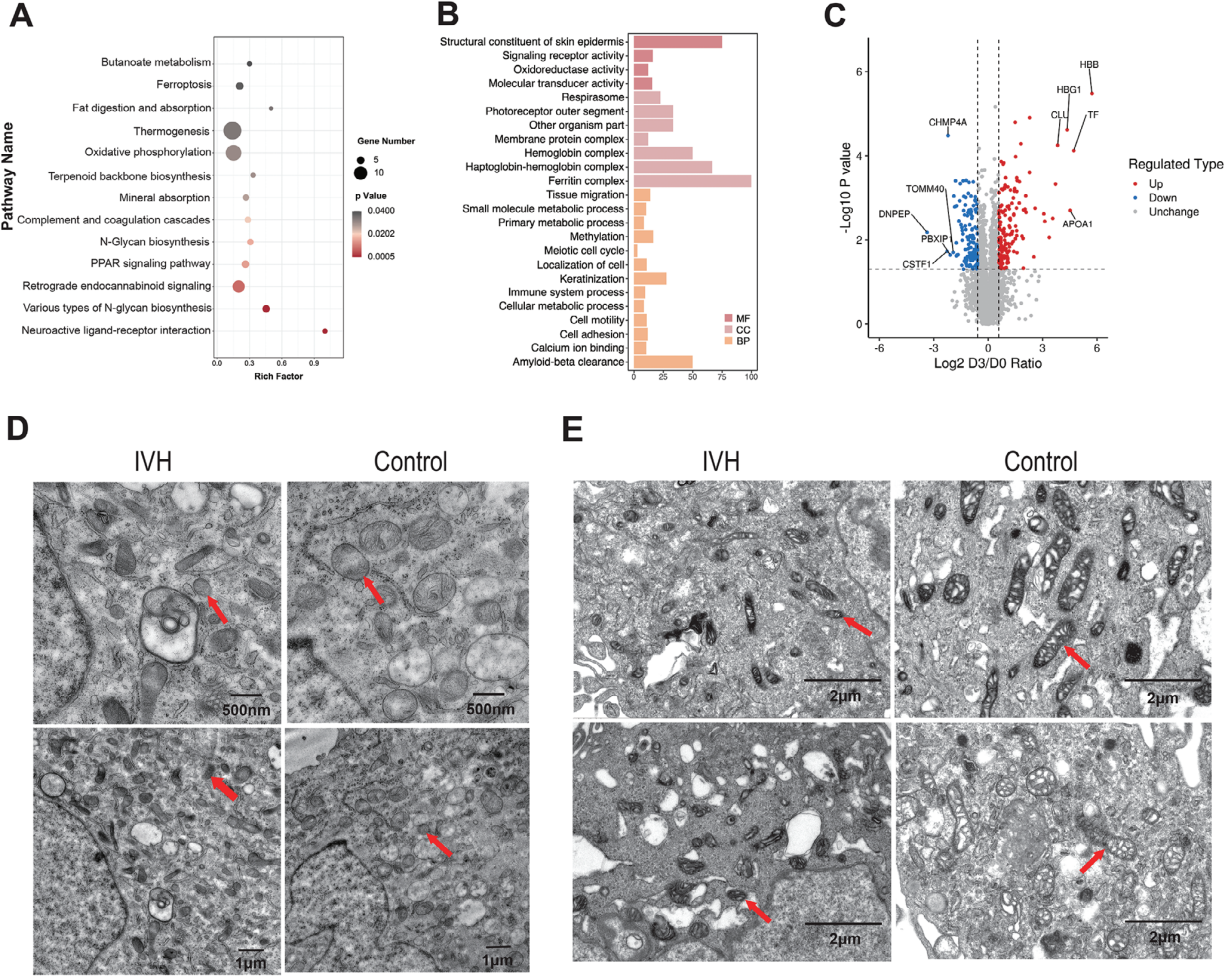
The proteomic profiling and results of transmission electron microscopy for cells treated with IVH‐CSF samples. A) KEGG‐based enrichment analysis for DEPs in IVH versus control group. B) GO‐based enrichment analysis for DEPs in IVH versus control group. C) Volcano plots show the protein alterations in HCPEpiCs treated with IVH‐ CSF samples (10 v/v %). The cells were incubated for 72 h. Proteins with |log_2_ (FC)| > 1.5 with an adjusted *P *< 0.05 were considered as potential DEPs. D,E) Transmission electron microscopy demonstrated mitochondria shrank, the mitochondrial membrane density increased, and the mitochondrial cristae broke down in HCPEpiCs (D), or pericytes (E) in the IVH group. HCPEpiCs, human choroid plexus epithelial cells; IVH, intraventricular hemorrhage.

The ChP is composed of epithelial, perivascular, and ependymal cells.^[^ ^24^ ^]^ To model IVH‐induced cellular changes in the ChP leading to PHH, ex vivo IVH CSF was used to stimulate human choroid plexus epithelial cells (HCPEpiCs) and primary human neurovascular pericytes; CSF of healthy infants (H‐CSF) was applied in control group.^[^ ^25^ ^]^ First, we performed LFQ–MS based proteomic analysis of HCPEpiCs stimulated with IVH‐ or H‐CSF samples. The KEGG pathways and GO molecular function analysis of DEPs showed that a considerable number of enriched biological processes were related to ferroptosis, such as fat digestion and absorption, oxidative phosphorylation, and the ferritin complex (Figure **4**A,B). Further information on LFQ‐MS results is summarized in Figure 4C; Table S4 (Supporting Information). Combined with the results of CSF biochemical data, metabolomics, proteomics, and HCPEpiC proteome sequencing, we found that pathways related to ferroptosis, calcium ion binding (and Ca^2+^) and cell adhesion were significantly activated. Therefore, we speculated that these pathways may be critical in the pathophysiology of PHH. We tested this hypothesis through the following experiments.

**Figure 5:**
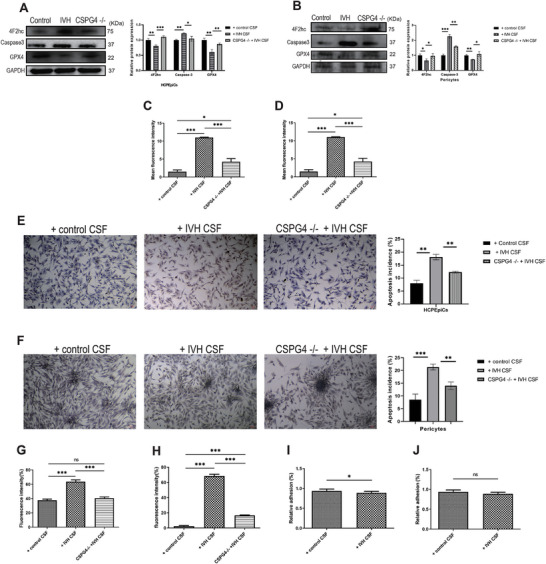
The analysis of ferroptosis, intracellular flow of Ca^2+,^ and cell adhesion in HCPEpiCs and primary human neurovascular pericytes treated with IVH CSF samples (10 v/v %). A,B) The protein levels of ferroptosis biomarkers in HCPEpiCs (A) or pericytes (B) treated with IVH‐CSF samples or lentiviral CRISPR/Cas9‐CSPG4‐KO construct; CSPG4, Chondroitin sulfate proteoglycan 4; GPX4, glutathione peroxidase 4; 4F2hc, lymphocyte activation antigen 4F2 large subunit. C,D) The results of reactive oxygen species assay in HCPEpiCs (C) or pericytes (D) treated with IVH‐CSF samples or lentiviral CRISPR/Cas9‐CSPG4‐KO construct. E,F) The results of TUNEL staining in HCPEpiCs (E) or pericytes (F) treated with IVH‐CSF samples or lentiviral CRISPR/Cas9‐CSPG4‐KO construct. G,H) The results of intracellular free Ca^2+^ in HCPEpiCs (G) or pericytes (H) treated with IVH‐CSF samples or lentiviral CRISPR/Cas9‐CSPG4‐KO construct. I,J) The results of cell adhesion function in HCPEpiCs (I) or pericytes (J) treated with IVH‐CSF samples or lentiviral CRISPR/Cas9‐CSPG4‐KO construct. Data was analyzed using one‐way ANOVA test. All data were obtained from three independent experiments. * *P* < 0.05; * * *P* < 0.01; * * * *P* < 0.001; ns, no significant; bars represent the mean ± s.e.m. IVH, intraventricular hemorrhage.

Transmission electron microscopy (TEM) results showed that the mitochondria shrank, the mitochondrial membrane density increased, and the mitochondrial cristae broke down in both HCPEpiCs and primary human neurovascular pericytes after IVH‐CSF stimulation (Figure 4D,E), which was the features of ferroptosis. The western blot (WB) results showed that glutathione peroxidase 4 (GPX4) and 4F2 cell‐surface antigen heavy chain (4F2hc) expression levels were significantly decreased in the IVH‐CSF treated group, comparing with those in the H‐CSF treated group; in contrast, caspase‐3 expression levels were significantly increased (Figure **5**A,B). The reative oxygen species (ROS) assay demonstrated that the mean ROS level was increased in the IVH‐CSF treated group (Figure 5C,D). Furthermore, Terminal Deoxynucleotidyl Transferase dUTP Nick End Labeling (TUNEL) staining showed a surprisingly higher incidence of cell death in the IVH‐CSF treated group (Figure 5E,F). All of these results confirmed that CSF from IVH patients be able to elicit ferroptosis in the HCPEpiCs and primary human neurovascular pericytes by measuring intracellular free calcium in HCPEpiCs and primary human neurovascular pericytes, the effect of IVH‐CSF on calcium ion binding was indirectly evaluated. The results showed that the level of intracellular free Ca^2+^ was significantly increased in both HCPEpiCs and primary human neurovascular pericytes treated with IVH‐CSF (Figure 5G,H). We next investigated the adhesion properties of HCPEpiCs and primary human neurovascular pericytes that were incubated with IVH‐CSF in vitro. The adhesive potential of HCPEpiCs treated with IVH‐CSF decreased to 85% of that was treated with H‐CSF, although in primary human neurovascular pericytes, there was no significant difference between two groups (Figure 5I,J).

**Figure 6:**
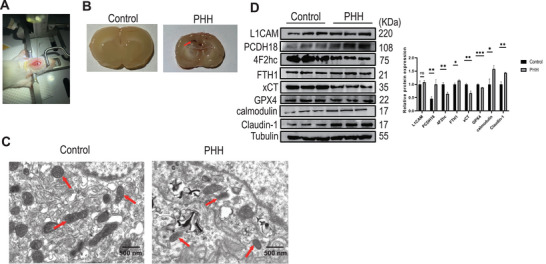
The rat model of PHH shows ferroptosis, cell adhesion, and calcium ion binding were abnormally activated in the choroid plexus. A) Schematic of a rat model of PHH was generated by stereotactically guided injection of autologous arterial blood. B) Schematic showed ventricular dilatation, and the white arrows indicate the lateral ventricles. C) Transmission electron microscopy showed the mitochondrial cristae became shorter, or even disappeared in cells in the PHH group. D) Western blot analysis of the biomarkers of ferroptosis (GPX4, 4F2hc, and xCT), calcium ion binding (PCDH18 and calmodulin), and cell adhesion (Claudin‐1 and L1CAM) in the choroid plexus of control rats and PHH group. Choroid plexus lysates (*n* = 3 rats) were collected and subjected to SDS–PAGE and to immunoblotting with the indicated antibodies. Data was analyzed using one‐way ANOVA test. All data were obtained from three independent experiments. * *P* < 0.05; * * *P* < 0.01; * * * *P* < 0.001; ns, no significant; bars represent the mean ± s.e.m. L1CAM, L1 cell adhesion molecule; PCDH18, Protocadherin 18; xCT, cystine‐glutamate transporter; 4F2hc, lymphocyte activation antigen 4F2 large subunit. PHH, post‐hemorrhagic hydrocephalus.

Next, a PHH rat model was generated by injecting autologous arterial blood into the ventricle (Figure **6**A,B). TEM results showed that the mitochondrial cristae became shorter, or even disappeared in the ChP cells of PHH rats, which was consistent with in vitro cellular characteristics, demonstrating ferroptosis (Figure 6C). Futhermore, the biomarkers of ferroptosis (GPX4, 4F2hc, and cystine‐glutamate transporter [xCT]), calcium ion binding (protocadherin 18 [PCDH18] and calmodulin), and cell adhesion (Claudin‐1 and L1 cell adhesion molecule [L1CAM]) were tested by WB analysis. The results demonstrated that whereas PCDH18, calmodulin, and claudin‐1 expression levels were dramatically elevated, whereas GPX4, 4F2hc, and xCT expression levels were significantly reduced in the ChP cells of PHH rat model (Figure 6D).

In summary, these in vitro and in vivo results confirmed that the activity of pathways of ferroptosis, calcium ion binding, and cell adhesion was altered in the ChP of PHH and suggested that these were the critical pathways involved in the pathophysiology of PHH.

### 2.6 *CSPG4* Silencing Inhibited Ferroptosis, Abnormal Cell Adhesion Function, and Intracellular Flow of Ca^2 +^in the Cellular Model

**Figure 7:**
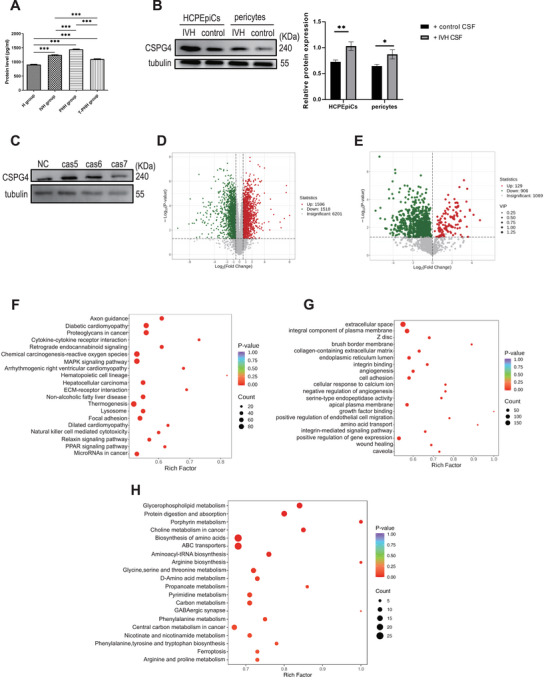
*CSPG4* silencing inhibits cell ferroptosis, abnormal adhesion function, and intracellular flow of Ca^2+^ progression. A) Enzyme‐linked immunosorbent assay (ELISA) validation of CSPG4 proteins expression levels in CSF among H (*n* = 37), IVH (*n* = 30), PHH (*n* = 52), and T‐PHH groups (*n* = 6). Data was analyzed using two‐tailed *t‐*tests. B) The protein levels of CSPG4 were decreased in HCPEpiCs and primary human neurovascular pericytes treated with IVH‐CSF samples, determined using Western blotting. C) The protein levels of CSPG4 were decreased in HCPEpiCs treated with lentiviral CRISPR/Cas9‐CSPG4‐KO construct. Data was analyzed using one‐way ANOVA test in C and D. All data were obtained from three independent experiments. D,E) Volcano plots show the proteins (D) or metabolite (E) alterations in HCPEpiCs treated with lentiviral CRISPR/Cas9‐CSPG4‐KO construct. F) KEGG‐based enrichment analysis for DEPs in HCPEpiCs treated with lentiviral CRISPR/Cas9‐CSPG4‐KO construct. G) GO‐based enrichment analysis for DEPs in HCPEpiCs treated with lentiviral CRISPR/Cas9‐CSPG4‐KO construct. H) KEGG‐based enrichment analysis for DEMs in HCPEpiCs treated with lentiviral CRISPR/Cas9‐CSPG4‐KO construct. * *P* < 0.05; * * *P* < 0.01; * * * *P* < 0.001; ns, no significant; bars represent the mean ± s.e.m. CSPG4, Chondroitin sulfate proteoglycan 4; HCPEpiCs, choroid plexus epithelial cells; H group, healthy preterm infants; IVH, intraventricular hemorrhage infants without hydrocephalus; PHH, post‐hemorrhagic hydrocephalus infants; T‐PHH; PHH infants who had serial LP therapy.

Based on the results of machine learning, Mfuzz analysis, and Pearson's correlation analysis, we selected SPP1, CHI31L, and CSPG4 for further study; however, the results of enzyme‐linked immunosorbent assays (ELISA) showed that the expression trends of SPP1 and CHI3L in the CSF among four groups did not match the pattern of disease progression (Figure S4A,B; Supporting Information). CSPG4 is a key intermediate in nerve cells and is abnormally expressed in many neurological diseases; however, its role in PHH is still unclear.^[^ ^26^ ^]^ The proteomic analysis indicated that CSPG4 expression in the CSF was gradually upregulated from the IVH group to the PHH group, and then downregulated in the T‐PHH group. These results were further confirmed using ELISA (Figure **7**A). The level of CSPG4 in HCPEpiCs and primary human neurovascular pericytes were significantly increased with IVH‐CSF stimulation (Figure 7B). These results suggested that CSPG4 was involved in PHH progression.

A clustered regularly interspaced short palindromic repeats‐associated nuclease 9 (CRISPR‐Cas9) gene editing system was used to knock out *CSPG4* in HCPEpiCs (Figure 7C). Then, proteomics and metabolomics were performed on the *CSPG4* knockout (*CSPG4* ^−/−^) HCPEpiCs. Overall, 1596 proteins were upregulated and 1518 proteins were downregulated in the proteome, while 129 metabolites were upregulated and 906 metabolites were downregulated in the metabolome, *CSPG4^−/−^* versus *WT* HCPEpiCs (Figure 7D,E). KEGG pathway enrichment and GO molecular function analysis showed that calcium ion binding, and cell adhesion were significantly activated in the *CSPG4^−/−^* cells (Figure 7F–H).

Next, we investigated further whether ferroptosis, intracellular Ca^2+^ flow, and cell adhesion function in IVH‐CSF treated HCPEpiCs or primary human neurovascular pericytes were influenced by *CSPG4* knockout. The results showed that the GPX4 and 4F2hc expression levels were robustly increased in both *CSPG4^−/−^* cells (Figure 5A,B). *CSPG4^−/−^* also ameliorated the cell adhesion function of HCPEpiCs (Figure 5I). Additionally, ROS level (Figure 5C,D) and intracellular free Ca^2+^ level (Figure 5G,H) were reduced in *CSPG4^−/−^* HCPEpiCs or *CSPG4^−/−^* primary human neurovascular pericytes.

Collectively, these results suggested that low levels of CSPG4 may protect ChP cells against injury in the PHH by influencing ferroptosis, cell adhesion, and the intracellular flow of Ca^2+^.

## 3 Discussion

In this study, we analyzed the biochemical, proteomic, and metabolomic profiles of the CSF from four independent clinical cohorts. To our knowledge, this study is the first to report the proteomic and metabolomic profiles of the CSF from infants with PHH. We found resources characterizing changes in 1296 proteins, 191 metabolites, Ca^2+^, and protein levels in the CSF of patients with PHH, of which several proteins and metabolites were upregulated in the CSF of patients with PHH compared with those in patients with IVH or healthy patients, and the changes in these proteins and metabolites could be ameliorated by LPs treatment. Importantly, using machine learning algorithms, we identified CSPG4, CHI3L1, SPP1, cytosine, and 5‐amino valeric acid as the potent biomarkers for PHH. Furthermore, Mfuzz and Pearson's analysis indicated that CSPG4 and other molecules participated in the progression of PHH, positively correlated with ventricular size and the rate of PVL. Pathway enrichment analysis revealed an over‐representation of ferroptosis, calcium ion binding, and cell adhesion signaling pathways associated with PHH. Next, we proved that these signaling pathways were also activated in the ChP in PHH using in vitro cellular models and a rat model of PHH, while *CSPG4* silencing could suppress the dysregulation of ferroptosis, cell adhesion function, and intracellular flow of Ca^2+^.

### 3.1 Molecular Insights for the Pathogenesis of PHH

To date, the pathogenesis of PHH is poorly understood because of the lack of sampling at the disease site. Supposedly, multi‐omics analysis of the CSF can offer unique insights into the pathogenesis of PHH. Previous studies have identified proteomic and metabolomic signatures of the CSF in congenital and idiopathic normal pressure hydrocephalus.^[^ ^26^, ^27^ ^]^ However, these signatures were not applied to PHH. In this study, we recruited a cohort including H, IVH without hydrocephalus group, and PHH groups, which represented the continuous progression of PHH. Thus, altered proteins and metabolites, such as CSPG4 and phenylethanolamine, may reflect the occurrence of diseases.

Previous studies have suggested that PHH could be caused by primary and secondary brain injury; thus, no one pathway or mechanism can fully explain PHH.^[^ ^28^, ^29^ ^]^ Our findings support this viewpoint.

Following GM–IVH, red blood cell lysis can release substances that break down red blood cells, particularly iron and ion pathway proteins, which are neurotoxic and can cause ferroptosis in nerve cells.^[^ ^30^ ^]^ Previous studies have shown that higher CSF ferritin levels were associated with larger ventricle size and poor neurodevelopmental outcomes in patients with PHH;^[^ ^31^, ^32^ ^]^ however, our results did not confirm this correlation using Pearson's analysis. Excessive iron produces ROS and leads to ferroptosis, which is implicated in neurological injury in IVH.^[^ ^33^, ^34^ ^]^ Our work buttressed these findings by bioinformatics analysis of CSF, in vitro cellular experiments, and a rat model of PHH.

Ca^2+^, a vital substance for preserving the stability of numerous signaling pathways, is crucial for normal physiological regulation of the central nervous system. Our study also revealed that Ca^2+^ levels were critical for PHH development. As mentioned previously, calcium–mediated proteolysis may be associated with axonal cytoskeletal damage in patients with hydrocephalus.^[^ ^35^ ^]^ Previous studies have also shown that dysfunction of the calcium‐binding protein Cetn2 could lead to hydrocephalus.^[^ ^36^ ^]^ Our study also found that the calcium‐binding proteins calmodulin and PCDH18 were significantly upregulated in the ChP of PHH. Therefore, our investigation suggested that abnormal calcium ion‐binding proteins and Ca^2+^ may be potential pathological mechanisms of PHH.

Numerous studies have shown that disruption of cell adhesion molecules could lead to hydrocephalus in adults and rats.^[^ ^37^, ^38^, ^39^, ^40^ ^]^ We also observed that cell adhesion molecules (claudin‐1 and L1CAM) were abnormally upregulated in the ChP of PHH rats. Subsequent investigations confirmed this using in vitro cellular experiments with aberrant adhesion function, HCPEpiCs, rather than neurovascular pericytes.

CSPG4, also known as neuron‐glialantigen 2 (NG2), is essential for cell survival, migration, and angiogenesis.^[^ ^41^, ^42^ ^]^ C.S.Carter et al. revealed that the aberrant development of NG2**^+^** PDGFRα**^+^** neural progenitor cells could lead to neonatal hydrocephalus.^[^ ^43^ ^]^ B. Ojeda‑Perez et al. found that the higher levels of the NG2 antigen were over‐expressed in the cerebrum of hydrocephalic mice.^[^ ^44^ ^]^ Our research confirmed that CSPG4 was involved in the development of PHH via the regulation of ferroptosis, cell adhesion function, and intracellular flow of Ca^2+^, which was in line with these previous studies.

### 3.2 Insights for PHH Therapeutics

To date, several studies have assessed the CSF of infants with PHH versus infants with IVH to explore disease biomarkers and potential targets (matrix metalloproteinases 2, amyloid precursor protein, and neural cell adhesion molecule 1, *etc*) for nonsurgical intervention; our proteomic data also showed that these proteins were upregulated in the PHH group (Table S2, Supporting Information). However, the sample sizes in most of these studies were small, which may warrant validation in larger studies.^[^ ^45^, ^46^, ^47^ ^]^

Here, we proposed that pathways of ferroptosis, abnormal adhesion function, and calcium ion binding were activated in PHH using bioinformatics analysis, cellular experiments, and a rat model of PHH; these signaling pathways have also been reported in previous studies.^[^ ^6^, ^35^, ^40^ ^]^ Interestingly, *CSPG4* silencing inhibits cell ferroptosis, abnormal adhesion function, and the intracellular flow of Ca^2+^ progression. These findings strongly suggested that patients with PHH might benefit from suppression of CSPG4.

In summary, our findings provide a highly valuable multi‐omics data resource for CSF from patients with PHH and multiple control groups for the research community to better understand PHH. We demonstrated the potential of identifying infants with IVH without hydrocephalus who may eventually develop PHH, based on an analysis of a panel of CSF proteins and metabolites. Importantly, our data offered a landscape view of the CSF molecular changes in infants with PHH who received LPs therapy, which may provide useful therapeutic agents for the treatment of the disease. Finally, we verified these results using in vitro cellular experiments and a rat model of PHH. In conclusion, this study has important clinical implications for patients with PHH.

## 4 Experimental Section

### Patient Information

The human and animal study protocols were approved by the Research Ethics Committee of the Guangzhou Women and Children's Medical Centre of Guangzhou Medical University and Dongguan Maternal and Child Health Hospital (No.2023021415102877). All parents were fully informed and signed written informed consent forms for this study.

Cranial ultrasound and MRI were performed in the infants from IVH, PHH, and T‐PHH groups; and all infants in the H group were also examined using cranial ultrasound. Infants with IVH without hydrocephalus group were those with low‐grade IVH (grades I–II), which was graded based on Papile's diagnostic criteria. Hydrocephalus was defined as ventriculomegaly with Evan's ratio (maximal width of frontal horns/maximal width of inner skull) >0.30 and/or lateral ventricles with ventricular width >97 th centile or anterior horn width >6 mm. Clinical data were collected individually from medical records. The CSF samples were collected between October 2019 and February 2021. All CSF samples were immediately frozen at 80 °C until use after being centrifuged at 3,000 g for 10 min to remove cells and debris.

### Label‐Free Quantitative Proteomics

Protein digestion and labeling were performed using a previously optimized protocol with minor modifications.^[^ ^48^ ^]^ CSF samples (10 µL) were added to a 6 m guanidine hydrochloride solution containing 1% protease inhibitor, then they were submerged in an ice bath and ultrasonically processed for 15 min. The mixtures were then reduced with 1 mol L^−1^ dithiothreitol for 1.5 h at 56 °C, and alkylated with 2 mol L^−1^ iodoacetamide in darkness for 30 min at room temperature. Then the protein mixture was further digested overnight at 37 °C in 50 mmol L^−1^ ammonium bicarbonate with trypsin. After digestion, the peptides were purified using SDB‐RPS StageTips, and loaded for mass spectrometry analysis. Peptides were dissolved in a mixture of 80% acetonitrile and 0.1% formic acid. Then the peptides were injected into an Eksigent nano High Performance Liquid Chromatography (HPLC) system. LC‐MS/MS analysis was performed using an EASY‐nLC 1200‐nanometer UHPLC system coupled with a Q Exactive TM Plus hybrid quadrupole‐orbitrap mass spectrometer (Thermo Fisher Scientific).

### Metabolomic Analysis

CSF samples (50 µL) were mixed with water and methanol (volume ratio 1:3) and vortex‐mixed for 30 s. The mixtures was vigorously shaken for 1 min. Protein precipitation was kept in a −20 °C freezer for 20 min, then samples were centrifuged at 14,000 g at 4 °C for 10 min to separate precipitated protein from extracted metabolites. Ultra‐performance liquid chromatography (UPLC)–MS/MS was used for untargeted metabolomic analysis. The UPLC–MS/MS methods used the ACQUITY 2D UPLC system and Q Exactive HF hybrid Quadrupole‐Orbitrap with HESI‐II heated ESI source and Orbitrap mass analyzer. The resulting MS/MS data were processed using Compound Discovery 2.0 search engine. The tandem mass spectra were searched against the BioCyc Human Metabolome database.

### Bioinformatic Analysis

Proteomic and metabolomic data were analyzed following three steps: 1) PHH group versus IVH group, 2) IVH + PHH group versus H group, and 3) PHH group versus T‐PHH group. Analyses with >50% missing values or near zero variances were excluded. The filter criterion for proteins and metabolites with significant differences in quantification between the groups was | Log2 fold change| > 1.5 and *P* < 0.05. KEGG analysis was performed for pathway, GO analysis was performed for molecular function, and cluster analysis was performed on the profile using the Mfuzz package. To construct proteins and metabolites interaction networks, the Wu Kong platform was used for proteomic and metabolomic joint pathway enrichment analysis, which was based on the ConsensusPathDB‐human database. The associations between the clinical information and DEPs (or DEMs) were examined using Pearson's correlation coefficients for categorical data. Estimations of ventricular size were calculated using the frontal/occipital horn radio [FOHR = (A+B) · 2C^−1^, A = bi‐frontal horn width, B = bi‐occipital horn width, and C = interparietal diameter].

### Machine Learning

Logistic regression, xgboost, lasso regression, rpart, support vector machine, random forest, and ranger models were used for predictions. The primary cohort was randomly divided into a development set (70%) and an internal validation set (30%). The bootstrap method was applied 1000 times to the internal validation set to estimate confidence intervals for the AUC, accuracy, sensitivity, and specificity. To optimize the hyperparameters for each model, a grid search approach with ten‐fold cross‐validation was employed. The permutation importance score of features, a widely used metric for assessing feature importance, was incorporated into the biomarker selection process. The criteria for selecting potential biomarkers were: 1) a *P*‐value of inter‐group difference ≤ 0.05, and 2) a permutation importance score ≥ 0 under the model with the maximum AUC.

### ELISA

The expression levels of CSPG4 in the CSF of the four groups were measured using a double‐antibody sandwich kits. The operation process was performed according to the manufacturer's instruction. The absorption value (optical density value) was measured at A450 using a microplate spectrophotometer (Bio‐Rad), and the expression level was calculated using standards.

### Treatment of Cells with the CSF Samples

HCPEpiCs were cultured in a special Epithelial Cell Medium (ScienCell); primary human neurovascular pericytes were cultured in a special pericytes edium supplemented with pericyte growth supplement (ScienCell); the procedures for cell culture followed the manufacturer's instructions. Ex vivo high‐grade GM–IVH (grades III and IV) or non‐IVH control CSF samples (10 v/v %) from preterm infants were added to the cell cultures when the cells reached ≈90% confluence; subsequently, the cells were incubated for 72 h. After incubation, the cells were washed with PBS, then harvested using a cell extraction buffer.

### Establishment of CSPG4 Knockout Cell Lines

The lentiviruses were packed by Genechem (Shanghai, China). The CRISPR/Cas9 system was used to introduce *CSPG4* deletions into the cells. Cas9 and single‐guide RNAs targeting the genomic *CSPG4* (Target DNA sequence: TCTCCACGGTGTCGTAAGCG) were designed using CRISPR and cloned into the GV708 vector. Cells were seeded into six‐well plates in complete medium and infected with the CRISPR/Cas9 virus for 48 h. EGFP‐positive cells were sorted by FACS. Western blotting analysis was used to determine the effectiveness of *CSPG4* knockout.

### Transmission Electron Microscopy

Glutaraldehyde (2.5%) in cacodylate buffer was used to fix the samples. After washing, the samples were post‐fixed in 2% osmium tetroxide for 2.5 h and dehydrated using a graded series of alcohols (50–70–95–100%), then infiltrated using propylene oxide for 1 .5h. Samples were embedded in EPON resin and hardened at 60 °C for 48 h. Sections were cut with a UC6 ultramicrotome, followed by a post‐staining for 15 min. A JEM‐100CX‐II transmission electron microscope was used for imaging.

### Intracellular Free Calcium Analysis

Fluo‐4 labeling of cells was used to measure intracellular calcium levels, according to the manufacturer's guidelines. Briefly, cells cultured on collagen‐coated confocal dishes were incubated with 4 µm Fluo‐4 AM in culture media containing 1% FBS at 37 °C for 40 min. After that, Hank's Balanced Salt Solution was used to wash the cells three times. Using flow cytometry, intracellular calcium levels was quantified.

### Cell Adhesion Assays

To quantify the adhesion function of cells treated with the CSF from patients with IVH, crystal violet staining was performed. Crystal violet (1 mL, 0.1%) was added to each well for 30 min. After removing the staining buffer, and the wells were washed again with PBS. Absorbance was measured at 540 nm using a microplate reader.

### TUNEL Staining

To detect apoptosis in HCPEpiCs and pericytes that were treated with the CSF from patients with IVH, TUNEL assay kit (Roche Applied Science, Sweden) was used according to the manufacturer's instructions. Apoptosis was observed using an inverted fluorescence microscope.

### ROS Assay

2′,7′‐dichlorofluorescein diacetate (DCFH‐DA) staining (Beyotime Biotechnology) was used to measure the ROS levels according to the manufacturer's instructions. In brief, the cells were digested with trypsin, and washed with PBS, then incubated with 10 µm DCFH‐DA in DMEM for 30 min at 37 °C. Fluorescence was measured by flow cytometry.

### The Rat Model of PHH

A rat model of PHH was generated according to a reported protocol with minor modifications.^[^ ^49^ ^]^ Simply, on the postnatal 10‐day pups were anesthetized. The rat was then placed in a stereotactic device, and a midline scalp incision was made to reveal the skull. A high‐speed drill was then used to create a 1 mm burr hole. Twenty‐five microliters of newly drawn autologous blood were collected from the tail artery, free from anticoagulants, and was infused into the right lateral ventricle (coordinates, x = −1.0, y = −0.5, z = −3.4 mm from bregma). The control condition was an intraventricular infusion of sterile aCSF administered in the same manner.

### Western Blotting

The following primary antibodies were used: anti‐calmodulin(CST, MA, USA), anti‐caspase‐3(CST), anti‐CSPG4 (CST), ferroptosis antibody sampler kit (CST), anti‐LAMP1 (CST), and anti‐PCD18 (SAB, USA), and β‐tubulin antibody (CST).Total protein was extracted from the cells with RIPA lysis buffer. Polyvinylidene fluoride membranes were used to transfer the proteins after they had been separated using with 10% sodium dodecyl sulfate‐polyacrylamide gel electrophoresis. After that, block the membranes with 5% milk in TBS/0.05% Tween‐20 for 1 h at room temperature. The bound proteins were then incubated with primary antibodies overnight at 4 °C. After extensive washing with TBST, the membranes were incubated with horseradish peroxidase‐conjugated secondary antibodies for 1 h. Image J software was used to determine the protein band intensities through densitometric analysis.

### Statistics

Data were analyzed by using SPSS version 22 and GraphPad Prism 10.5. Means (standard deviations or standard error of mean) or medians (range) were used to describe continuous variables; two‐tailed *t‐*tests, one‐way ANOVA, or Kruskal–Wallis tests were used to analyze the differences in continuous variables. A two‐sided chi‐square test or Fisher's exact test was used for categorical variables, which are presented as numbers and percentages. *P* values < 0.05 were considered a statistically significant difference.

## Conflict of Interest

The authors declare no conflict of interest.

## Author Contributions

M.L., M.Z., and W.Z. contributed equally to this work and shared the correspondence authorship. J.C., W.Z., M.Z., and M.L. designed the study, designed the data collection instruments, collected data, carried out the initial analyses, drafted the initial manuscript, and created the tables and figures; L.W., X.P., T.C., and Y.Y., conceptualized and designed the study, coordinated and supervised data collection, and helped draft the initial manuscript; J.S., H.Z., S.W., Y.M., L.W., and X.Y. designed the data collection instruments, collected data, conducted the initial analyses; All authors contributed to the manuscript's critical revision and read and approved the submitted version.

## Supporting information



Supporting Information

## Data Availability

The data that support the findings of this study are available from the corresponding author upon reasonable request.
