# Supplementary material for: Identification of CSPG4 as a Biomarker and Therapeutic Target for Infantile Post‐Hemorrhagic Hydrocephalus via Multi‐Omics Analysis
Source: Adv Sci (Weinh). 2024 Dec 16;12(6):2410056. doi: 10.1002/advs.202410056 (PMC11809374; doi:10.1002/advs.202410056)
Supplement: Supplementary file 1 — Supporting Information [file ADVS-12-2410056-s001.docx]

**Identification of CSPG4 as a Biomarker and Therapeutic Target for Infantile Post-Hemorrhagic Hydrocephalus via Multi-Omics Analysis**

Juncao Chen, Lin Wang, Xiangwen Peng, Tingting Chen , Yihui Yang, Jingzhen Su, Hongmei Zuo, Siyao Wang, Yueting Mao, Lixiang Wu, Xuntao Yin, Minxu Li ^*^, Mingwei Zhu^*^ , Wei Zhou^*^

**Supplemental Tables**

**Table S1. Top 10 differentially expressed proteins in the PHH+ IVH vs. H group, PHH vs. IVH group, PHH vs. T-PHH group**.

|  | **Protein** | **FC** | ***P* value** |
| --- | --- | --- | --- |
| PHH+IVH *vs*. H |  |  |  |
|  | FTL | 10.01 | 7.45 × 10^-10^ |
|  | CSTB | 4.93 | 9.95 × 10^-9^ |
|  | CHI3L1 | 10.05 | 2.88 × 10^-8^ |
|  | HTRA1 | 7.31 | 1.17 × 10^-7^ |
|  | LTF | 3.51 | 6.28 × 10^-7^ |
|  | RPS3 | 0.57 | 6.56 × 10^-7^ |
|  | HEBP1 | 5.33 | 3.33 × 10^-6^ |
|  | PLD3 | 1.40 | 4.41 × 10^-6^ |
|  | **WFIKKN2** | 0.43 | 6.05 × 10^-6^ |
|  | SPP1 | 3.71 | 9.85 × 10^-6^ |
| PHH *vs*. IVH |  |  |  |
|  | SPP1 | 4.67 | 1.94 × 10^-8^ |
|  | CHI3L1 | 7.77 | 1.86 × 10^-7^ |
|  | **RNASET2** | 5.31 | 5.99 × 10^-7^ |
|  | **FCGR3A** | 6.86 | 5.99 × 10^-7^ |
|  | FTL | 3.82 | 8.90 × 10^-7^ |
|  | CSTB | 2.05 | 1.38 × 10^-6^ |
|  | **PLA2G7** | 8.81 | 2.03 × 10^-6^ |
|  | **ADA2** | 6.03 | 2.96 × 10^-6^ |
|  | **CD84** | 4.04 | 3.73 × 10^-6^ |
|  | **MERTK** | 4.31 | 5.91 × 10^-6^ |
| PHH *vs.*T-PHH |  |  |  |
|  | **PVR** | 0.50 | 6.06 × 10^-3^ |
|  | **SPP2** | 1.82 | 6.06 × 10^-3^ |
|  | **SHBG** | 1.85 | 7.63 × 10^-3^ |
|  | IGKV2-30 | 2.86 | 9.56 × 10^-3^ |
|  | **AEBP1** | 1.57 | 1.28 × 10^-2^ |
|  | **SDF4** | 1.78 | 1.58 × 10^-2^ |
|  | **CAPN1** | 1.66 | 1.70 × 10^-2^ |
|  | **CACNA2D3** | 2.35 | 1.70 × 10^-2^ |
|  | **NUDT5** | 2.17 | 1.82× 10^-2^ |
|  | **TMEFF1** | 2.21 | 1.95× 10^-2^ |
| FC, fold change; *p* values were adjusted for multiple comparisons by the Benjamini–Hochberg Method. | | | |

**Table S2. Some differentially expressed proteins and metabolites in various clusters according to the Mfuzz analysis.**

|  | **Proteins and Metabolites** | |
| --- | --- | --- |
| Cluster 1(n=55) | | ALB, AFB, NELL2, FBLN1, APLP1, CHGA, AFM, CST3, CRTAC1, KNG1, PKM, SEZ6, PGLYRP2, DAG1, LMAN2, VCAN, PSAP, NPC2, GFRA2, 1-Methylguanine, Kanosamine, L-Tryptophan, Cytosine, DL-3-Aminoisobutyric acid,1-Methylguanine |
| Cluster 2(n=99) | | HSP90AA1, MYH9, ACTN4, ACTN1, PGK1, SPARC, MPO, PDIA3, GPI, EEF1A1, VCP, PGD, CAP1, ENO2, EPRS1, MST1, FBLN2, RNH1, PNP, CORO1A,PC 17:1_17:1, DL-Dipalmitoylphosphatidylcholine, PC 18:2_18:2, PC 18:0_20:5, PC 19:2_19:2, PC 18:0_18:1 |
| Cluster 3(n=76) | | CFH,APOA4,IGHG1,SPARCL1,C1S,ITIH4,SCG3,IGHG2,IGHG3,SCG2,VGF,  OGN,AMBP,NID2,HRG,IGKC,APOD,LAMA2,HPR,SEZ6L, Creatinine, N-Butylbenzenesulfonamide, Isoleucine, 2E)-3-phenyl-N-(2-phenylethyl)prop-2-enamide, 2-Hydroxycaproic acid |
| Cluster 4(n=46) | | COL1A1,TUBA1A,KRT8,QSOX1,PABPC1,WDR1,CPB2,PCSK9,IGLV3-19,  FLNC,IGHV4-4,AKR1C2,IGLV3-10,HDGF,BMP1,IGKV2-30,IGLV8-61,EPHB2,FRRS1L,NDST1,RARRES2,IGHG1,LPC18:1,1-Methyladenosine,  N-Acetyl-L-carnosine, SM 8:1;2O/34:3, LPC 22:4-SN1 |
| Cluster 5(n=68) | | CP,F2,SERPINA3,C2,EFEMP1,SERPINA7,RELN,GAP43,BASP1,HINT1,PON1,RPS3,NPDC1,MRC2,CD55,MCAM,MEX3C,XPO1,GAA,DSG2,OAF,IGHV2-70D, N-Butylbenzenesulfonamide, PC 17:0_18:5, Urocanic acid,SM 8:1;2O/25 |
| Cluster 6(n=109) | | APOB ,HSP90AB1,HSPA8,IGHM,SPTB,KRT6A,CHI3L1,GAPDH,DSP,TUBB4B,IGHA1,YWHAE,SPP1,MMP9,HBZ,HTRA1,C1QB,FABP5,IGHA2,CPN2,CFD,KRT13, Phenylethanolamine,Indole-3-acetic acid, PC 15:0_16:0,  SM 8:0;2O/26:0 |
| Cluster 7(n=86) | | CHGB,APOA1,GSN,CPAMD4,HSPG2,CNTN1,TTR,COL6A3,CACNA2D1,SERPINF1,ITIH1,PTGDS,CDH2,ECM1,NEO1,APOH,EEF2,ORM2,CFHR1,SPON1, NTM, LY6H, SM 8:1;2O/34:1,Caprolactam, Prolylleucine,  N-Butylbenzenesulfonamide, Hypoxanthine |
| Cluster 8(n=68) | | TF,PLG,GC,FBLN1,HPX,APLP1,NID1,VIM,HP,DKK3,LGALS3BP,C6,IGFBP2,PCOLCE,CLEC3B,VTN,ORM1,WFIKKN2,IGFBPL1,IGLL5,LAMC1,PPIA,IGHV3-72, RTBDN,IGHV3-49,Caprolactam, Adenosine, 3-Hydroxyanthranilic acid, Sulfamethazine,1,3,7-trimethyl-2,3,6,7-tetrahydro-1H-purine-2,6-dione, |
| Cluster 9(n=205) | | C4B ,KRT1,ACTB,KRT2,HBA1,CLU,SPTA1,HBG2,CAT,HBB,TGFBI,ENO1,  KRT16,HBD,ALDOA,LTA4H,GDI2,CALR,FTL,YWHAB,GOT1,BPGM,PRDX1, FSTL1,C8B,MAN2A2, LGALS1,HBE1, PC 16:1_16:1,Kanosamine,Oleamide, PC 16:0_17:2, Diosgenin |
| Cluster 10(n=53) | | NRCAM,TKT,PLEC,TUBB,EIF4A1,ANXA5,SHBG,NCL,ANXA1,SLC3A2,H2BC18,H2AC25, G6PD,TYMP,HSPA9,PAPLN,CAPS,HGFAC,EIF5A,DRAXIN,  IGLV3-9, HSPE1, PC 18:1_20:4, PC 16:0_20:4,PC 16:0_20:3,PC 18:0_18:2, HexCer 18:2;3O/15:0;(2OH) |
| Cluster 11(n=67) | | FLNA,FASN,TLN1,ACLY,CCT5,HNRNPA1,H4C1,PRNP,YWHAG,FDPS, RACK1,TPM4,HNRNPL,IGHV5-10-1,CNDP2,PA2G4,SERPINI1,PROC,CTSH,ARPC1B,HMGB1,IGLV5-39, CHST10,PC 18:0_20:5,PC O-38:6,PC O-38:5,PC 19:1_19:2, PC O-36:5, PC O-38:4,TKK |
| Cluster 12(n=36) | | TUBA1C,KRT6B,COL3A1,LMNA,PTPRD,SPP1,IQGAP1,P4HB,S100A11,  ANXA3, YKT6,ERAP1, IGHV4-34,F13A1,ITGAM,LGALS3,PIGR,  CTSS,COLEC12,MDH2,C1QTNF3, PC O-32:2, SM 8:1;2O/28:0,  N-Butylbenzenesulfonamide, 13(S)-HOTrE, SM 8:1;2O/32:1,PC O-38:3 |
| Cluster 13(n=35) | | PKM,HSPD1,IGFBP7,RNASE1,FBN1,IGHV3-7,STIP1,FABP7,MRC1,PREP, TIPRL, RPS26P11, IGKV6D-21,LFNG,NSF,UBE2V2,F11,COL12A1,CNDP1,  CDH12,TPST1, SM 8:1;2O/25:0,Palmitoyl sphingomyelin,3-Pyridinol,  SM 8:1;2O/30:1, Sitagliptin |
| Cluster 14(n=168) | | RPS26P11,FN1,SERPINA1,CHL1,C7,SERPINC1,POSTN,CLSTN1,AHSG,  NCAM1,AGT,APP,MEGF8,B4GAT1,IGHG4,AGRN,ROBO1,CDH11,CADM1,  S100A9,LSAMP,F12,NFASC,GFRA2,CDH5,Trigonelline,  Kanosamine,(5Z)-3-aminonon-5-enoic acid, Ecgonine, LNAPE 20:4/N-20:0,  PC 18:0_22:4 |
| Cluster 15(n=207) | | FGB,FGA,HRNR,FGG,KRT9,LTF,HBG1,ENPP2,NCAN,BCAN,PLTP,ACTC1,PTPRS,YWHAZ,SEMA7A,SOD1,TMEM132A,OPCML,CTSB,CCT7,PRDX6,LMNB1,VWF,PTPRZ1, PC 17:0_18:2,  PC 16:0_16:1,Solanine,5-Methylcytosine,octadec-9-ynoic acid, Palmitoleic Acid, |
| Cluster 16(n=109) | | C4A,A2M,FCGBP,KRT10,APOE,ITIH2,KRT14,PROS1,FLG2,AZGP1,LRP1,UBA1,B2M,IGLC3,PLXDC2,GDI1,TIMP2,SOD3,GOLM1,CADM3,PLXNB2,CAPG,5-Aminovaleric acid, Choline, Hypoxanthine, L-(+)-Lactic acid |

|  |
| --- |

**Table S3. Top 10 differentially expressed metabolites in the PHH+ IVH *vs.* H group, PHH *vs.* IVH group, PHH *vs.* T-PHH group.**

|  | **Metabolite** | **FC** | ***P* value** |
| --- | --- | --- | --- |
| PHH+IVH *vs.* H |  |  |  |
|  | Cytosine | 0.32 | 1.55× 10^-9^ |
|  | PC O-40:7 | 2.22 | 2.20× 10^-9^ |
|  | PC O-32:0 | 3.41 | 6.49× 10^-9^ |
|  | PC O-34:1 | 1.60 | 3.95× 10^-8^ |
|  | Diosgenin | 3.66 | 2.42× 10^-7^ |
|  | PC O-44:5 | 6.38 | 2.55× 10^-7^ |
|  | PC O-36:4 | 1.69 | 6.52× 10^-7^ |
|  | PC O-16:1_16:0 | 1.79 | 7.54× 10^-7^ |
|  | PC O-18:1_16:0 | 1.65 | 9.14× 10^-7^ |
|  | D-(+)-Tryptophan | 2.96 | 9.59× 10^-7^ |
| PHH *vs.* IVH |  |  |  |
|  | 5-Aminovaleric acid | 1.94 | 7.68× 10^-7^ |
|  | Cytosine | 0.50 | 3.25× 10^-6^ |
|  | PC O-37:7 | 0.51 | 1.01× 10^-5^ |
|  | Hypoxanthine | 1.35 | 7.45× 10^-5^ |
|  | Diosgenin | 2.61 | 7.75× 10^-5^ |
|  | 5-Methylcytosine | 1.77 | 1.64 × 10^-4^ |
|  | PC O-32:0 | 1.75 | 2.69× 10^-4^ |
|  | Palmitic Acid | 0.56 | 4.66× 10^-4^ |
|  | Oleamide | 1.25 | 6.66 × 10^-4^ |
|  | Prolylleucine | 2.00 | 9.12 × 10^-4^ |
| PHH *vs*.T-PHH |  |  |  |
|  | PC O-37:7 | 2.69 | 4.78 × 10^-3^ |
|  | Palmitoyl sphingomyelin | 0.06 | 1.19 × 10^-2^ |
|  | PC 20:3_20:3 | 1.51 | 2.09 × 10^-2^ |
|  | PC O-32:2 | 0.68 | 3.74 × 10^-2^ |
|  | 1-Methylguanine | 3.69 | 3.75 × 10^-2^ |
|  | LPA 16:0 | 0.42 | 5.72 × 10^-2^ |
|  | HexCer 18:2;3O/15:0;(2OH) | 1.52 | 8.03 × 10^-2^ |
|  | 1-Methyladenosine | 0.53 | 8.48 × 10^-2^ |
|  | 1,3,7-trimethyl-2,3,6,7-tetrahydro-1H-purine-2,6-dione | 1.85 | 9.44 × 10^-2^ |
|  | SM 8:1;2O/30:1 | 0.12 | 9.45 × 10^-2^ |

FC, fold change; *p* values were adjusted for multiple comparisons by the Benjamini–Hochberg method.

**Table S4. Top 10 differentially expressed proteins in human choroid plexus epithelial cells treated with IVH- CSF *vs.* H-CSF samples (10 v/v %).**

| **Protein** | **FC** | ***P* value** |
| --- | --- | --- |
| HBB | 52.74 | 3.31 × 10^-6^ |
| FTH1 | 4.88 | 1.25 × 10^-5^ |
| H1-0 | 2.80 | 1.60 × 10^-5^ |
| HBG1 | 20.43 | 2.40× 10^-5^ |
| CHMP4A | 0.22 | 3.32× 10^-5^ |
| DAP | 3.48 | 5.17× 10^-5^ |
| CLU | 14.20 | 5.65× 10^-5^ |
| TF | 26.25 | 7.54× 10^-5^ |
| ZNF638 | 2.85 | 1.05× 10^-4^ |
| GRIPAP1 | 1.65 | 1.36× 10^-4^ |
| FC, fold change; *p* values were adjusted for multiple comparisons by the Benjamini–Hochberg method. | | |

**Supplemental Figures**


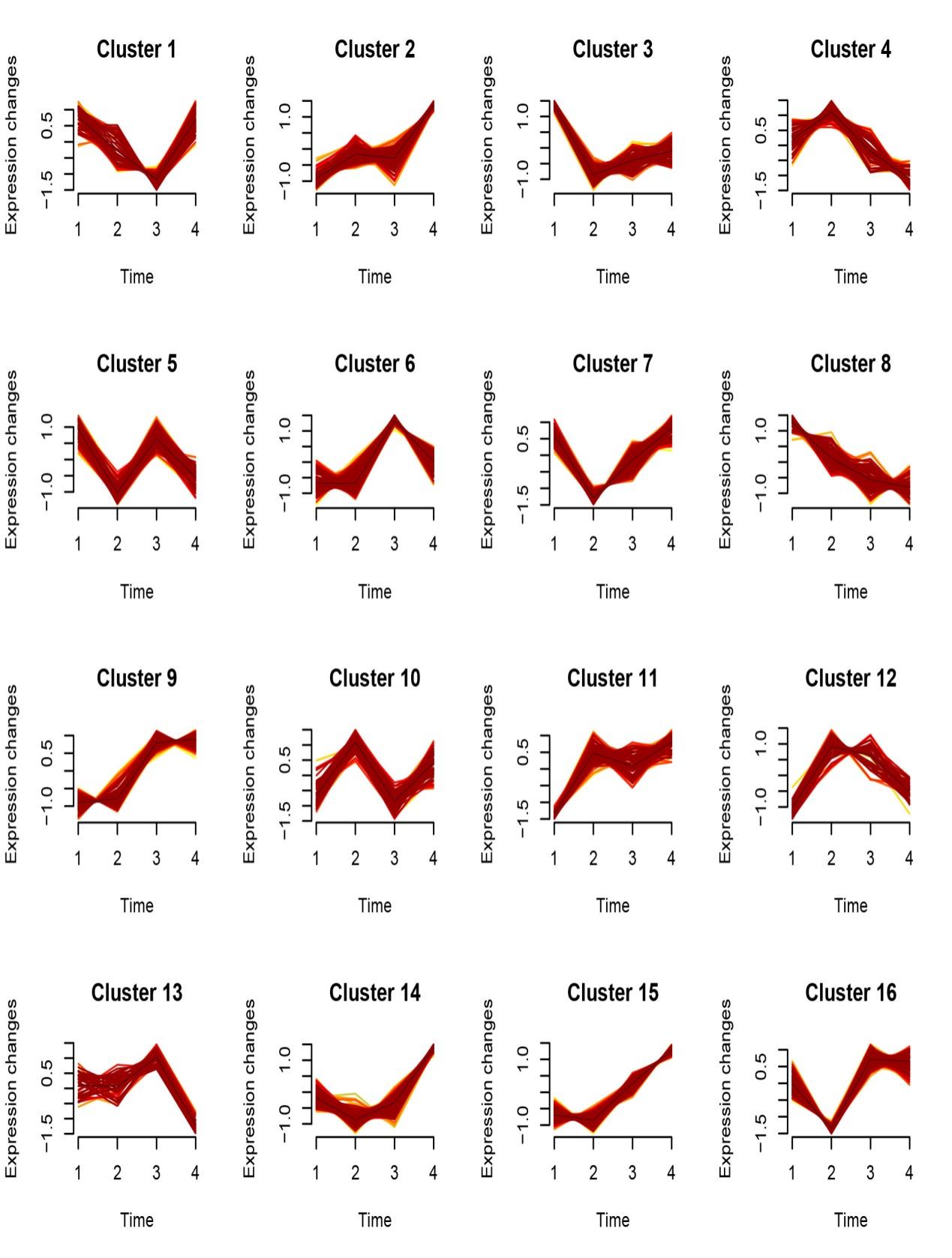


**Figure S1**. **Identification of specific clusters of proteins and metabolites among 4 groups of patients by the Mfuzz analysis.** 1: Healthy infants group; 2: IVH group; 3: PHH group; 4: T-PHH group. H group, healthy preterm infants; IVH, intraventricular hemorrhage infants without hydrocephalus; PHH, post-hemorrhagic hydrocephalus infants; T-PHH; PHH infants who were treated with serial LP therapy.


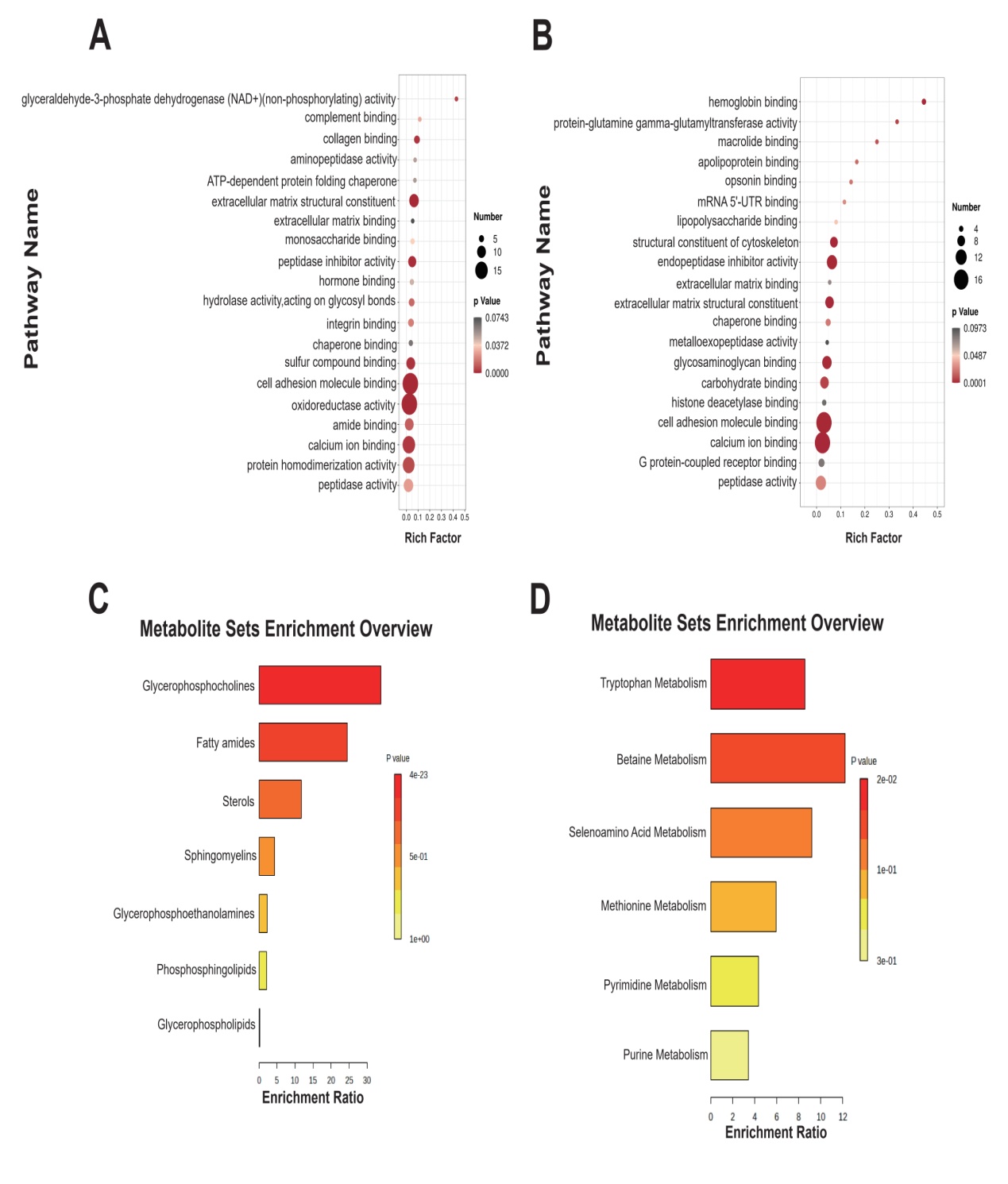


**Figure S2. Continuously increasing or decreasing proteins and metabolites both in IVH vs. H and PHH vs. IVH groups. (A) KEGG integration analysis of continuously increasing proteins both in IVH vs. H and PHH vs. IVH groups.** (B) KEGG integration analysis of continuously decreasing proteins both in IVH *vs.* H and PHH *vs.* IVH groups. (C) KEGG integration analysis of continuously increasing metabolites both in IVH *vs.* H and PHH *vs.* IVH groups. (D) KEGG integration analysis of continuously decreasing both metabolites in IVH *vs.* H and PHH *vs.* IVH groups. H group, healthy preterm infants; IVH, intraventricular hemorrhage infants without hydrocephalus; PHH, post-hemorrhagic hydrocephalus infants.


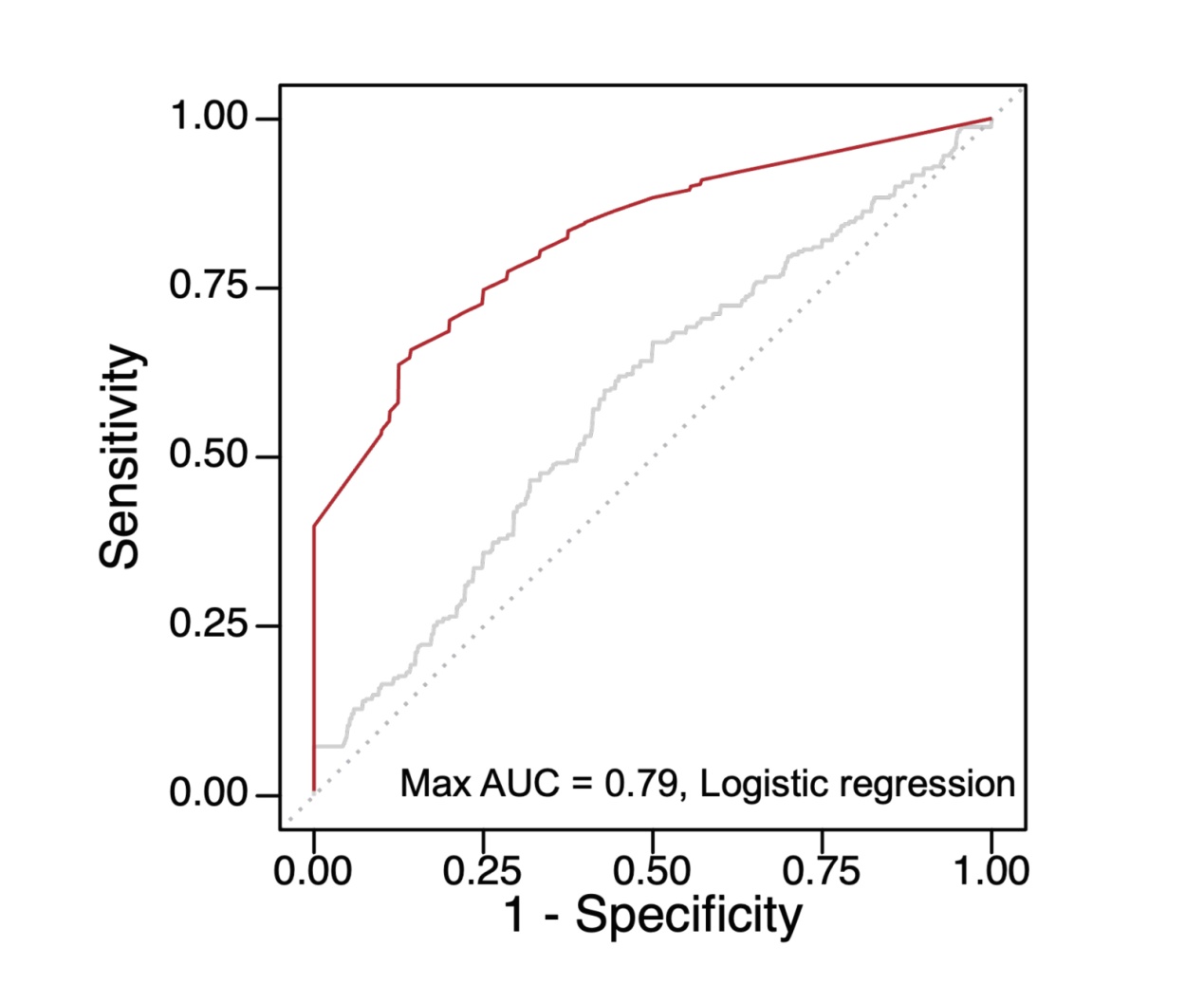


**Figure S3. Machine learning model showed that CSPG4 could be a biomarker for PHH.**


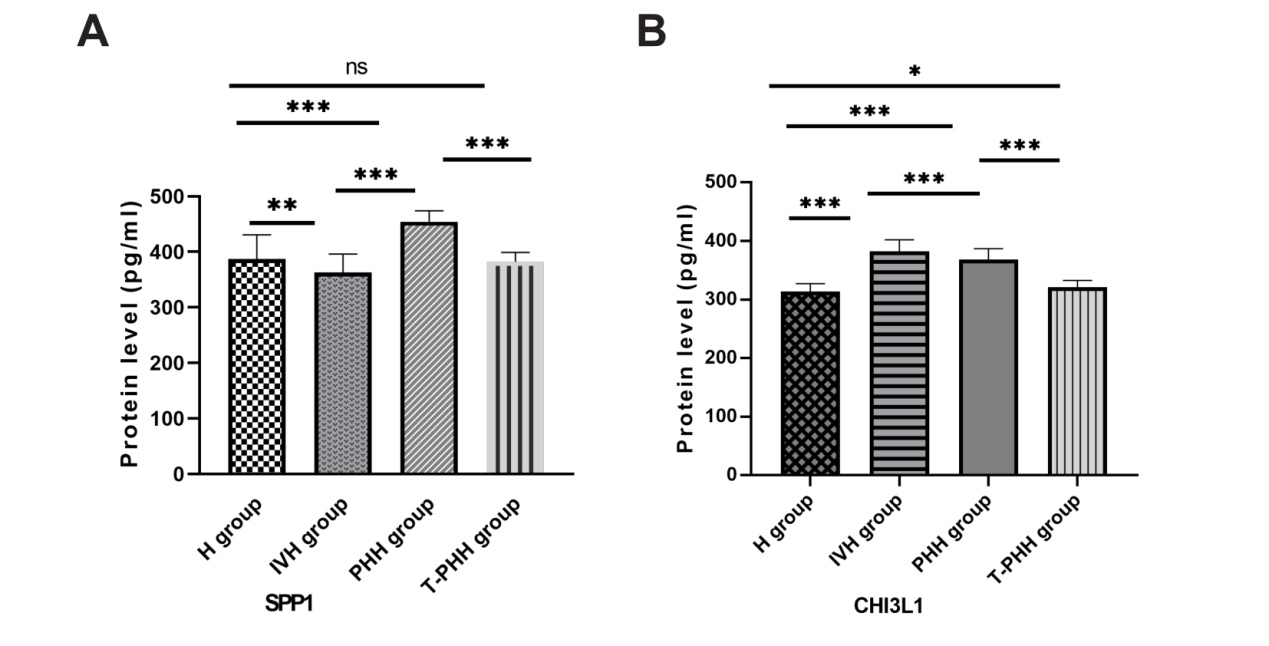


**Figure S4.** **Enzyme-linked immunosorbent assay (ELISA) validation of SPP1(A) and CHI3L(B) proteins expression levels in CSF among H ( n=37 ), IVH ( n=30), PHH ( n=52), and T-PHH groups (n=6).** Data was analyzed using two-tailed *t-*tests. * *P* < 0.05,* * *P* < 0.01, * * * *P* < 0.001, ns; no significant. H group, healthy preterm infants; IVH, intraventricular hemorrhage infants without hydrocephalus; PHH, post-hemorrhagic hydrocephalus infants; T-PHH; PHH infants who were treated with serial LP therapy.
